# Expression and New Exon Mutations of the Human Beta Defensins and Their Association on Colon Cancer Development

**DOI:** 10.1371/journal.pone.0126868

**Published:** 2015-06-03

**Authors:** Abdelhabib Semlali, Abdullah Al Amri, Arezki Azzi, Omair Al Shahrani, Maha Arafah, Muhammad Kohailan, Abdulrahman M. Aljebreen, Othman alharbi, Majid A. Almadi, Nahla Ali Azzam, Narasimha Reddy Parine, Mahmoud Rouabhia, Mohammad S. Alanazi

**Affiliations:** 1 Genome Research Chair, Department of Biochemistry, College of Science King Saud University, Riyadh, Kingdom of Saudi Arabia; 2 College of Medicine, King Saud University, Riyadh, Kingdom of Saudi Arabia; 3 Division of Gastroenterology, King Khalid University Hospital, King Saud University, Riyadh, Saudi Arabia; 4 Division of Gastroenterology, The McGill University Health Center, Montreal General Hospital, McGill University, Montreal, Canada; 5 Groupe de Recherche en Écologie Buccale, Département de stomatologie, Faculté de Médecine Dentaire, Université Laval, Québec, Québec, Canada; 6 College of Medicine, Al Imam Muhammad Ibn Saud Islamic University (IMSIU), Riyadh, Kingdom of Saudi Arabia; CRCL-INSERM, FRANCE

## Abstract

The development of cancer involves genetic predisposition and a variety of environmental exposures. Genome-wide linkage analyses provide evidence for the significant linkage of many diseases to susceptibility loci on chromosome 8p23, the location of the human defensin gene cluster. Human β-defensins (hBDs) are important molecules of innate immunity. This study was designed to analyze the expression and genetic variations in hBDs (hBD-1, hBD-2, hBD-3 and hBD-4) and their putative association with colon cancer. hBD gene expression and relative protein expression were evaluated by Real-Time polymerase chain reaction (qPCR) and immunohistochemistry, respectively, from 40 normal patients and 40 age-matched patients with colon cancer in Saudi Arabia. In addition, hBD polymorphisms were genotyped by exon sequencing and by promoter methylation. hBD-1, hBD-2, hBD-3 and hBD-4 basal messenger RNA expression was significantly lower in tumor tissues compared with normal tissues. Several insertion mutations were detected in different exons of the analyzed hBDs. However, no methylation in any hBDs promoters was detected because of the limited number of CpG islands in these regions. We demonstrated for the first time a link between hBD expression and colon cancer. This suggests that there is a significant link between innate immunity deregulation through disruption of cationic peptides (hBDs) and the potential development of colon cancer.

## Introduction

Colorectal cancer (CRC) is the third most commonly diagnosed cancer among males and the fourth most common among females worldwide [1]. The American Cancer Society estimates there will be over 96,000 new cases of colon cancer leading to approximately 50,000 deaths in 2014 (**American cancer society 2014**). In the Kingdom of Saudi Arabia (KSA), colon cancer is one of the most frequent diseases [2], with a median age of 60 years for males and 58 years for females [3]. Cancer development has been reported to involve genetic factors [4] and also a variety of environmental exposures [5]. Genetic susceptibility to cancer is multifactorial, including somatic genetic alterations, such as mutations in oncogenes or tumor suppressor genes [6], and changes in gene expression profiling or DNA methylation. Gene expression profiling has offered a new way to classify human tumors [7]. Based on the mRNA expression levels of specific genes, different subtypes of cancer can be identified. DNA methylation is the most widely studied epigenetic marker [8]. DNA hypermethylation-induced gene silencing is a common event in many malignancies, serving as an alternative mechanism to genetic mutation to affect the loss of tumor suppressor functions [9,10]. The discovery of global DNA hypomethylation in human tumors was followed by the identification of hypermethylated tumor suppressor genes, and recently, inactivation of microRNA (miRNA) by DNA methylation has also been described [11,12]. This form of epigenetic change may contribute to tumor initiation and progression through transcriptional silencing of tumor suppressor genes. Indeed, several genes have been shown to be epigenetically inactivated ina wide range of tumors [13]; thus, the concept of a 'hypermethylation profile' of tumors can have potential clinical applications [14–16]. Hypermethylated genes may include those involved in cell cycle regulation (p16INK4a, p15,Rb) [17], DNA repair (BRCA1, MGMT), resistance (MGMT), cellular differentiation, angiogenesis (THBS1) and metastasis [13]. However, limited information exists about the role of the deregulation of innate immunity genes and their plausible association with colon cancer. Furthermore, evidence for the role of the natural immune system in protection from tumor development has been demonstrated by studies involving immuno-compromised patients [18]. It is well documented that a weak immune response has a direct and inverse correlation with many types of cancer [19,20]. All cells in the human body have multiple lines of defense against cellular transformation, and the human immune system is a wonderful and well-coordinated network of cells, organs, and glands that protects the body from inappropriate physiological deregulations. An optimized immune system is the key to good health and longevity. Furthermore, the innate immune response has considerable specificity against conserved molecular patterns of microorganism components, which are called pathogen-associated molecular patterns (PAMPs). The receptors on immune cells that recognize PAMPs are called pattern recognition receptors (PRRs). Toll-like receptors (TLRs) are a major class of PRRs, and each TLR recognizes a different PAMP [21–23]. Activation of an innate immune response proceeds though binding to pattern recognition receptors such as TLRs. These TLRs are key sensors of invading pathogens, largely activated by innate immunity actors such as epithelial cells [23]. The TLR signaling cascade may involve the activation of the adapter molecule MyD88, and both cascades lead to the activation of NF-κB to promote the transcription of pro-inflammatory cytokines, chemokines and cationic peptides also known as human beta-defensins (hBDs). These hBDs belong to a family of antimicrobial peptides that constitute an important part of the innate immune defense. To date, four hBDs (1–4) have been identified in human tissues. hBD-1 is constitutively produced by various epithelial tissues such as the respiratory tract and skin [24], whereas the expressions of hBDs are inducible [25]. Specifically, hBD-2 is highly expressed in normal epithelial cells following contact with microorganisms or cytokine (TNF- and IL-1) stimulation [26,27].

Their genomic structure consists of two exons and one intron. The first exon encodes the signal peptide, and the second encodes a mature peptide preceded by a short anionic pro-peptide. The mature peptide is obtained after the proteolytic cleavage of the signal sequence. Despite the low sequence similarities between these proteins, their 3D structures have a similar defensin-like topological fold consisting of three-strands arranged in three anti-parallel sheets constrained by three intra-molecular disulfide bridges [28,29]. Beta-defensins have a cluster of cationic residues (Lys, Arg) near the carboxyl termini of the peptides. The C-terminal positively charged amino acids play a determinant role in the antimicrobial activity [30,29]. The positive net charge and hydrophobic properties promote hBDs interaction with microbial membranes. hBDs exert direct antimicrobial action and are active against bacteria, fungi, and viruses; in parallel, according to recent data, they possess multiple biologic activities, in particular, immuno-modulatory ones [29], and are implicated in the anti-tumor response.

The objective of this study was to investigate the deregulation of the hBDs gene expression profile, hBDs exon mutations, and promoter methylation of hBDs as well as the association of these findings with colon cancer promotion in humans. From a clinical point of view, proteins that are involved in these pathways can serve as targets for cancer therapy, through the potential use of hBD/TLR-immunotherapy.

## Results

### Clinical data of patients diagnosed with colon cancer via colonoscopy

The clinical characteristics of 40 Saudi patients, including age, nationality, family history, smoking habits, stage of colon cancer, medications and presence of other diseases, were collected and compared between colon cancer and control patients. The enrolled population includes non-smokers with no family history of colon cancer and without allergies. The study population age ranged between 45 and 75 years, with a mean of 60 ±16.56 years for males and 55 ±13.74 years for females (Table 1). It is also interesting to note that a high number of the participants suffering from colon cancer were not undergoing chemotherapy or radiotherapy.

**Table 1:** Clinical data of patients diagnosed with colon cancer via colonoscopy.

**Gender**		Age	Nationality	Locality	Physical activity	Smoker/ Alcoholic	Family history	Therapy
Male	24	60 ±16.56	Saudi = 23 Non Saudi = 1	Colon = 7 Rectum = 6 Sigmoid = 7 Recto-Sigmoid = 2 Cecum = 1	Yes = 7 No = 17	Smoker = 0 NonSmoker = 17 Exsmoker = 7 Alcoholic = 1 Non alcoholic = 23	Yes = 7 No = 17	Chemotherapy No = 23 Yes = 1 Radiology No = 23 Yes = 1
Female	16	55 ±13.74	Saudi = 14 Non Saudi = 2	Colon = 5 Rectum = 2 Sigmoid = 8 Recto-Sigmoid = 1 Cecum = 0	Yes = 3 No = 13	Smoker = 0 NonSmoker = 16 Exsmoker = 0 Alcoholic = 0 Non alcoholic = 16	Yes = 4 No = 12	Chemotherapy No = 16 Yes = 0 Radiology No = 16 Yes = 0
Total	40	58 ±15.5	Saudi = 37 Non Saudi = 3	Colon = 13 Rectum = 8 Sigmoid = 15 Recto-Sigmoid = 3 Cecum = 1	Yes = 10 No = 30	Smoker = 0 NonSmoker = 0 Exsmoker = 7 Alcoholic = 1 Non alcoholic = 39	Yes = 11 No = 29	Chemotherapy = 0 Radiology = 1

### Differential hBD gene expression in colon cancer tissues

**Fig 1:**
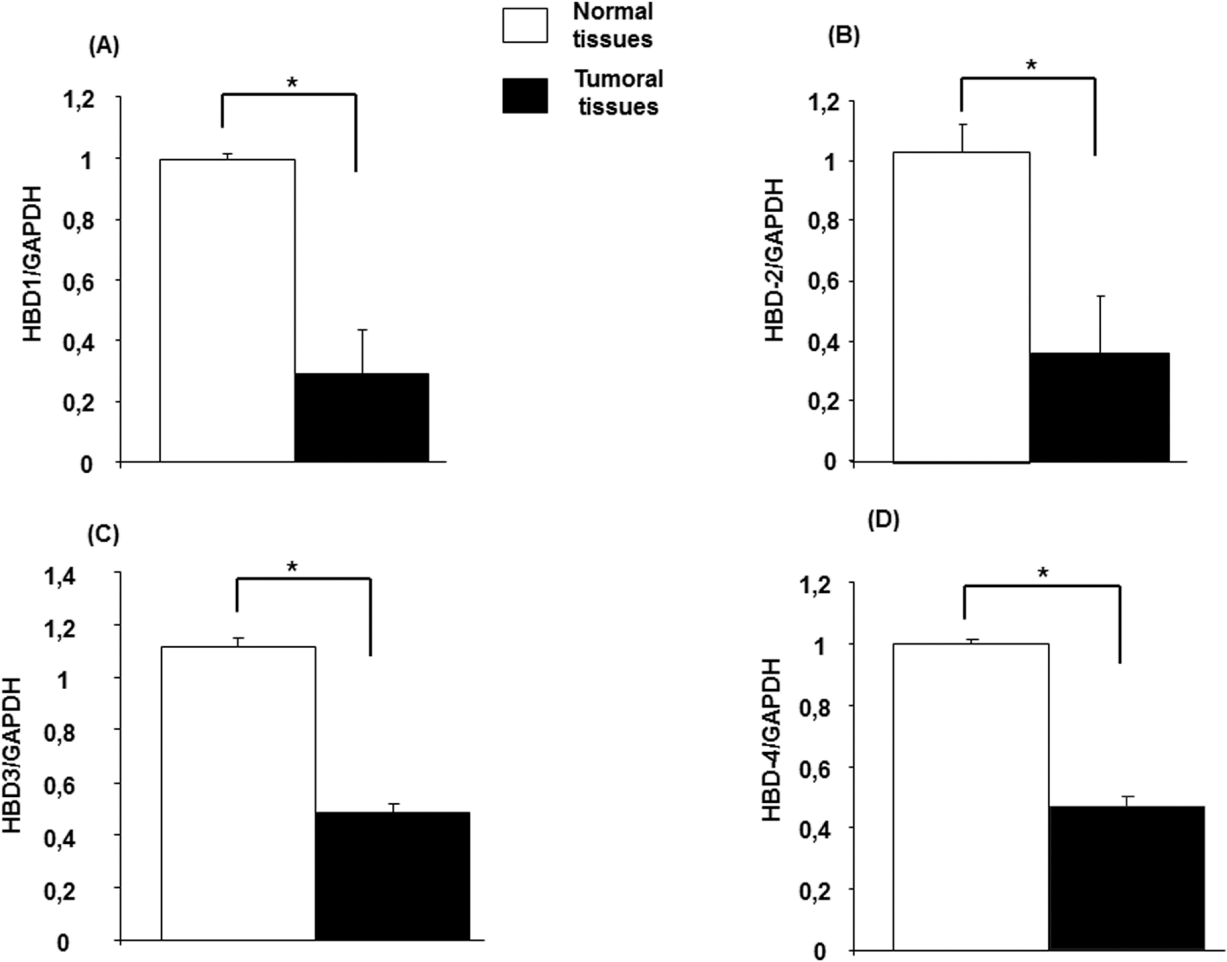
Human beta defensin (hBD) mRNA and protein expression in colon cancer tissues. Total cellular RNA freshly extracted from normal and colon cancer tissues was reverse transcribed into cDNA and then used to measure the hBD mRNA expression (Panel1A to 1D).

To analyze the expression of the different hBDs (1–4) at the mRNA level, quantitative real-time reverse transcription-PCR was performed using colon cancer tissues and normal tissues isolated from the same patient. As shown in **Fig 1,** the expression levels of all the hBDs were decreased in cancer tissues compared with normal tissues. Specifically, the hBD-1 levels decreased significantly (p < 0.0001) from 0.99 ± 0.02 in the normal tissues to 0.29 ± 0.14 in the cancer tissues (**Fig 1A)**. hBD-2 levels dropped from 1.03 ± 0.08 in the controls to 0.36 ± 0.18 in the cancer tissues, with p < 0.0001 (**Fig 1B)**. hBD-3 decreased from 1.11 ± 0.11 in the control tissue to 0.48 ± 0.09 in the cancer tissues (**Fig 1C)**, and hBD-4 dropped from 0.99 ± 0.03 in the control tissues to 0.46 ± 0.10 in the cancer tissues **(Fig 1D)**.

### Immunohistochemical comparison between different antimicrobial peptides

**Fig 2:**
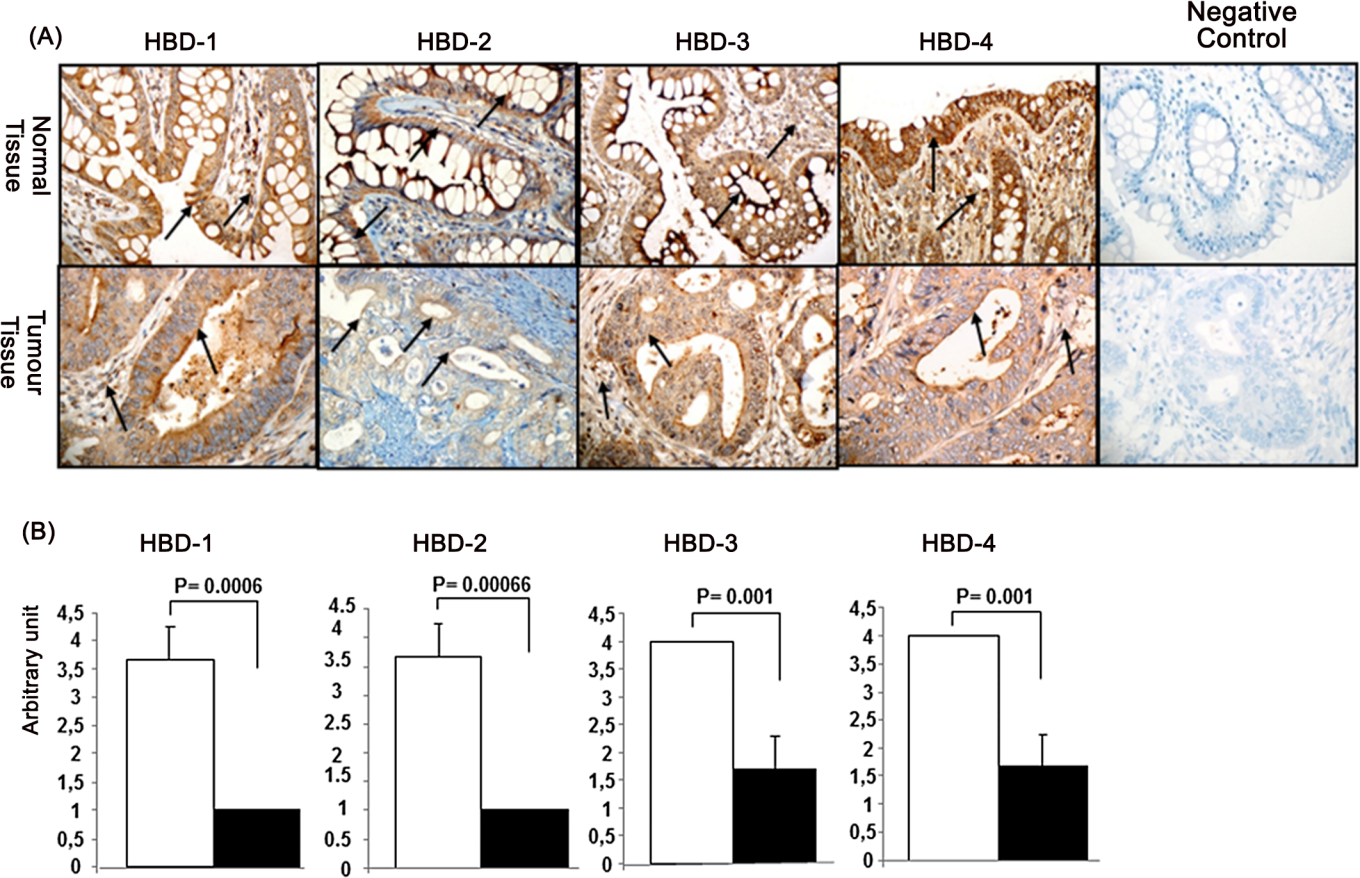
Human beta defensin (hBD) protein expression in colon cancer tissues. Tissues were immunostained using specific hBD antibodies (Panel 2A). hBD- positive cells in the tissues were estimated as follows: 0 points, no positive color; 1 point, <20% positive staining; 2 points, 21‑50% positive staining; 3 points, 51–75% positive staining; and 4 points, >75% positive staining. This is presented in Panel B.

To confirm the mRNA expression data, we determined the hBDs protein expression by immunohistochemistry. As shown in **Fig 2A,** positive immuno-staining for the hBDs was generally observed in normal colon epithelial cells and also in some stromal cells. However, the intensity of staining for all the hBDs was lower in the adenocarcinoma colon tumor tissues. In order to quantitatively determine the differential expression of hBDs in colon cancer tissues in comparison to normal tissues, we counted the positive cells and determined an arbitrary expression level, as presented in Fig 2B to 2D. These results confirmed the low levels of hBDs in cancer tissues compared with normal tissues. The observed low level of hBD proteins confirms the finding of low levels of hBD gene expression.

### Prevalence of HBD exon mutations in colon cancer patients

To investigate the association of hBD mutations and their lower expression in colon cancer patients, exon sequencing for all hBDs was analyzed. The *de novo* mutation rate of hBDs has been estimated in Table 2, for hBD-1; three mutations were detected in exon 1 (two in the 5’UTR region before the translated sequence and one in the promoter). These mutations do not affect the protein structure, but can influence the expression and stability of mRNA. Two other insertion mutations were detected in the translated region of exon 2 that led to an immature protein. We also found five mutations in the 3’-untranslated region (3'UTR). The 3'UTR is known to contain regulatory elements that are essential for the appropriate expression of several genes. Two types of mutations were detected: 8 (80%) were insertions and 2 (20%) were transitions. As indicated in Table 2, the same patient may contain one or more mutations in the hBD1 region (example T17 = colon cancer patient number 17 had 3 different mutations of the hBD-1 gene). The transition mutation in the 3'UTR of the hBD-1 gene is not associated with colon cancer, but is instead linked to the Saudi population, as it was found in all normal and cancer participants except in one case (T4).

**Table 2:** Summary of hBDs mutations and their nature/location found in colon cancer tissues.

Mutation number	Base change	Mutation type	Structural change	Region	Coding description	Number/ total Tumors	ID Sample
**hBD-1**							
1	CTGA>CTGAA	Insertion	No consequence	promoter	c.47 + A	3 /20	T8, T30 and T33
2	GCCT>GCCTA	Insertion	No consequence	5’UTR	c.67 + A	3 /20	T4, T5 and T8
3	CTGGG>CTGGA	Transition	No consequence	5’UTR	c.132 G>A	19/20	All samples except T4
4	AGG>AGGC	Insertion	No mature protein	Exon2	c.7182+ C	2 /20	T31 and T17
5	CAG>CAGA	Insertion	No mature protein	Exon2	c.7199+r A	3 /20	T17, T46 and T44
6	AGTG>AGTGC	Insertion	No consequence	3’UTR	c.8354 +C	3 /20	T46, T44 and T24
7	ATAA>ATAAAC	Insertion	No consequence	3’UTR	c.8371 +AC	3 /20	T46, T44 and T24
8	TAAT>TAATC or TAATA	Insertion	No consequence	3’UTR	c.8384 + C or A	2 /20	+C (T17 and T24)+A (T44 and T17)
9	GGAA>GGAAC	Insertion	No consequence	3’UTR	c.8391 + C	3 /20	T46, T44 and T24
10	AAGTA>AAGTC	Transition	No consequence	3’UTR	c.8407 A>C	3 /20	T46, T44 and T24
**hBD-2**							
1	TCAC>TCAG	Tranversion	No consequence	Flanking sequence	c.-72 C>G	2/20	T14 and T33
2	GCTG>GCTGC	Insertion	No consequence	Flanking sequence	c.-58+ C	2 /20	T16 and T33
3	TAATG>TAATA	Transition	No consequence	Flanking sequence	c.-39 G>A	3 /20	T23, T44 and T16
4	TGATG>TGATA	Transition	No consequence	3’UTR	c.1962 G>A	3 /20	T22, T44 and T46
5	ATGGA> ATGGG	Transition	No consequence	3’UTR	c.2069 A>G	All patients	All tumors and normals
**hBD-3**							
1	CCAGT>CCAGTA	Insertion	No consequence	5’UTR	c.191+A	3 /20	T11, T18 and T32
2	GGAT>GGATA	Insertion	Sequence change starting residue 4	Exon 1	c.262+ A	2 /20	T11 and T46
3	AATGC>AATGCT	Insertion	C63L, R64P, R65K, K67E	Exon 2	c.1347+T	2/40	T15 and T32
4	GAAA>GAAAA	Insertion	K67E	Exon 2	c.1358+A	4/40	T17, T18, T32 and T33
5	GAAAT>GAAATA or GAAATT	Insertion	No consequence Or No stop codon	Exon 2 near stop	c.1363+A or T	2 /20	T11 and T17
6	TGAC>TGACC	Insertion	No consequence	3’UTR	c.1382+C	3 /20	T11, T32 and T47
7	CGAG>CGAGA	Insertion	No consequence	3’UTR	c.1385+A	100% (N+T)	All tumors and normals
8	AGTGT>AGTGTG	Insertion	No consequence	3’UTR	c.1400 +G	100% (N+T)	All tumors and normals
**hBD-4**							
1	GCCC>GCCT	Transition	No consequence	5’UTR	c.7 C > T	6/20	T21, T22, T23, T30, T31and T33
2	ATTT>ATTTA	Insertion	Sequence change starting residue 22	Exon 2	c.4577 + A	2/20	T22 and T46
3	CCTAT>CCTATA	Insertion	Complete changes starting residue 56	Exon 2	c.4670 +A	5/20	T22, T33,T46, T15 and T44

N = Normal colon tissue

T = Tumor

In hBD-2, four total mutations were detected in colon cancer tissues, but not in normal tissues: three in the promoter (one transition mutation and two insertion mutations), no mutations in the translated sequences for exon 1 and exon 2, and two transition mutations in the 3’UTR region (one transition in the 3’UTR is not associated with colon cancer but with the Saudi population). In hBD-2, three types of mutations were detected: 1 (20%) was a transversion, 2 (20%) was an insertion and 3 (60%) were transition mutations. All mutations detected in hBD-2 have no consequence on the protein structure but can affect hBD-2 expression and gene stability. In hBD-3, all mutations (100%) detected were insertion mutations (Table 2): one insertion was detected in the 5’UTR region, one insertion was found in the translated sequence of exon 1, inducing a complete sequence change starting at residue 4, as well as three other mutations in the translated region of exon 2 (one of these insertions was near the stop codon), and three insertions were detected in the 3’UTR region of the hBD-3 gene. Insertions 7 and 8 were specific to the Saudi population and were not associated with colon cancer; all tumors and all normal tissues presented these mutations (Table 2). In hBD-4, three total mutations were detected: one in the 5’UTR that did not affect the hBD-4 protein structure and two in exon 2 that generate an incomplete sequence, with complete sequence change starting at residues 22 and 56. Two types of mutations were detected in hBD-4: 1(30%) was a transition and 2 (60%) were insertions (Table 2). However, no methylation in any hBDs promoters was detected because of the limited number of CpG islands in these regions.

### Structure—Function Analysis

**Fig 3:**
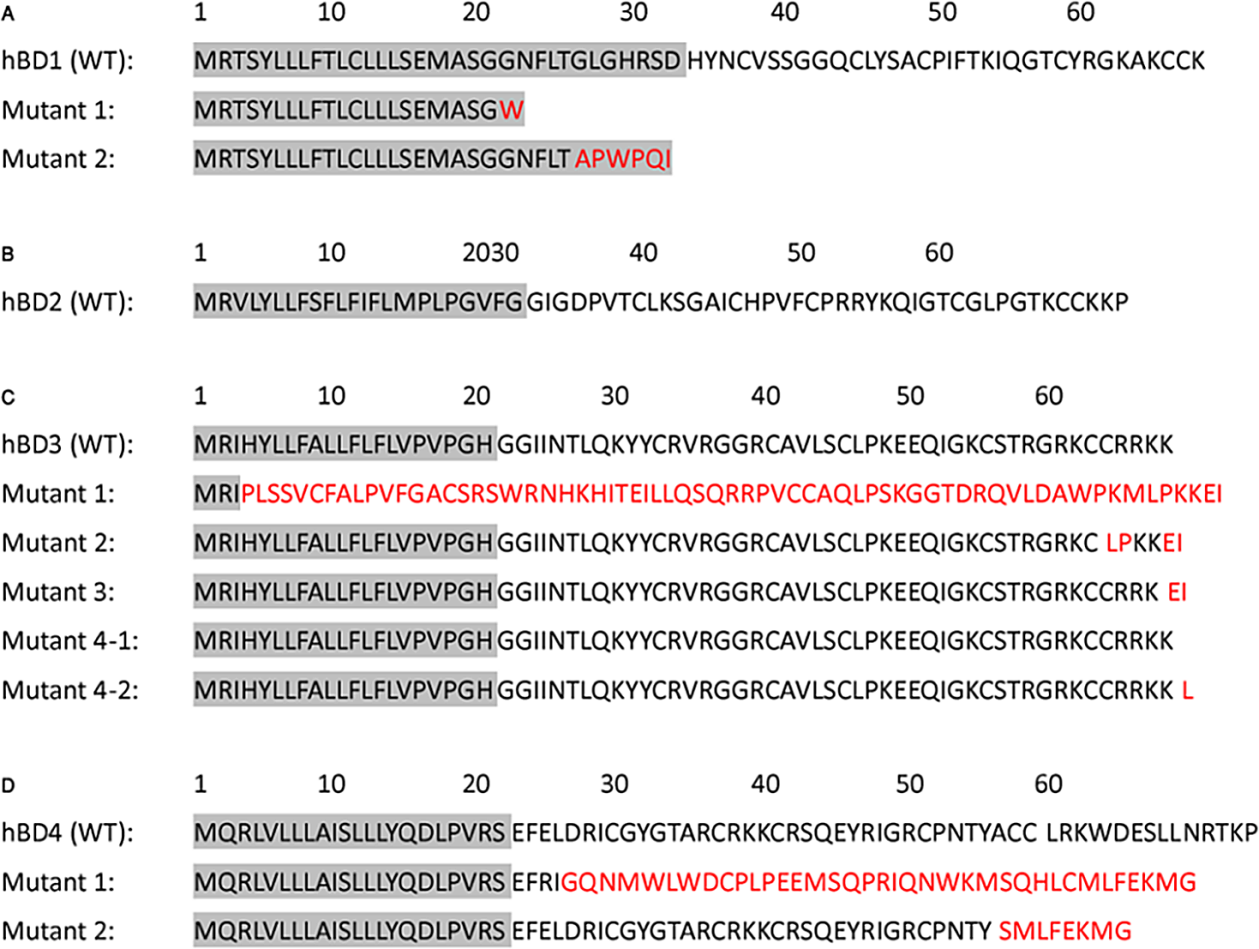
Amino acid sequence alignment of four human beta defensins (panel A for hBD1, panel B for hBD2,Panel C for hBD3 and panel D for hBD4) with the amino acid sequences for their corresponding observed mutants. Mutations are highlighted in red for each hBDs gene.

**Fig 4:**
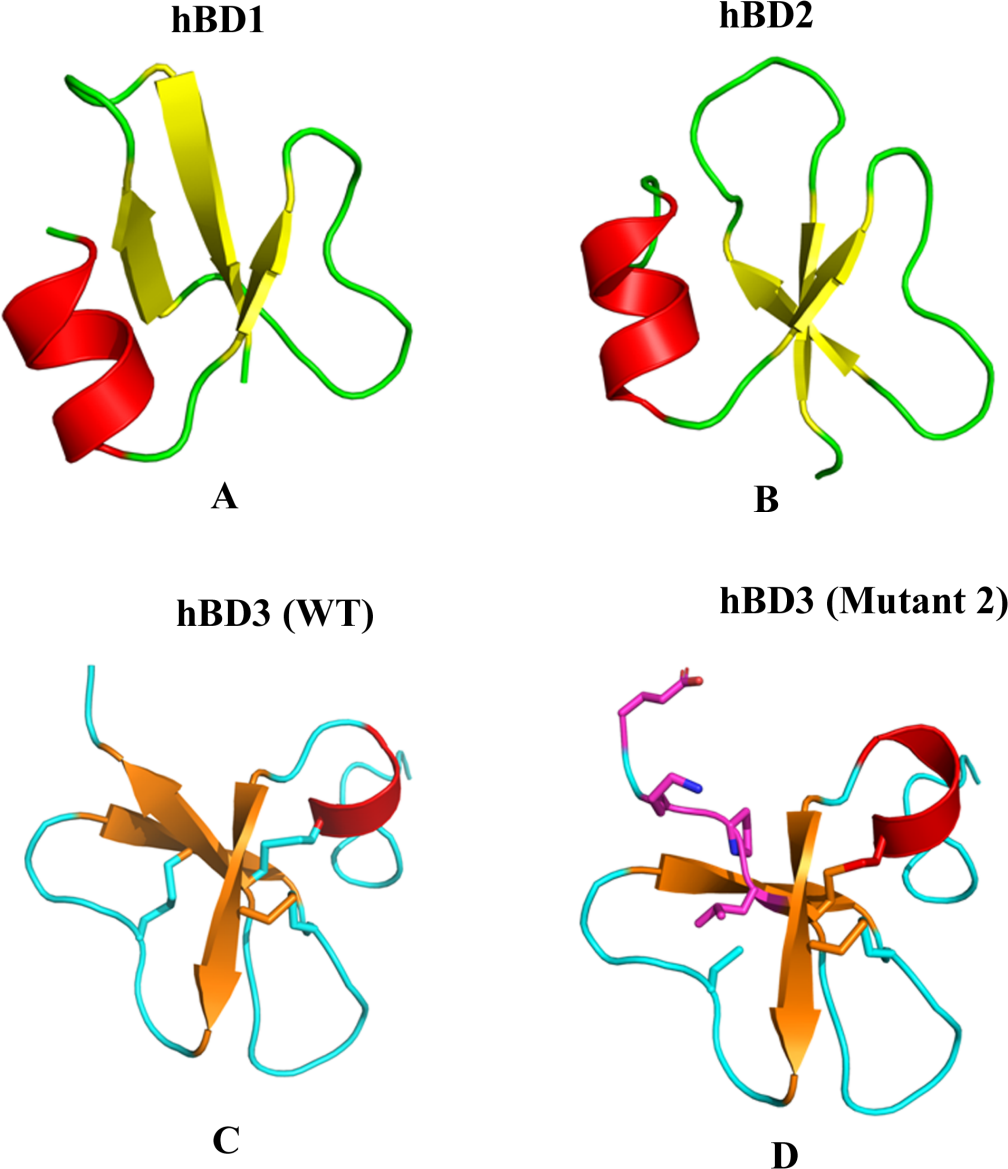
Differents structures of hBDs: hBD-1 (panel A), hBD2 (Panel B), and hBD3mutant and wild type (panel C and D).

We have examined the gene product of each exon affected by a frame-shift mutation as a consequence of the observed nucleotide insertions. The resulting sequence translations for each hBD with affected exon products are illustrated in Figs 3 and 4. The two insertions on exon 2 of hBD-1, as reported in Table 2, causes frame-shift mutations after the peptide signal sequence (Fig 3A). Consequently, no mature hBD1 protein can be synthesized. No insertion was found on exon 2 on the human hBD-2 gene (Figs 3B and 4B). Five insertions were found on the hBD-3 gene in colon cancer. Insertion 1 results in a frame-shift mutation from residue 3 in the peptide signal sequence, with no mature hBD-3 synthesis (Fig 3C). Insertion 2 causes a frame-shift mutation affecting 5 of 6 C-terminal residues, C63→L, R64→P, R65→K and K66→E (Fig 3C). Additionally, an inserted isoleucine is found in the sequence of the mutated hDB-3 at the C-terminal end (Fig 3C). To evaluate, the consequence of the mutation on the structure of hBD-3, we built a hBD-3 molecular model with the mutations affecting the C-terminal end. Fig 4C and 4D, illustrates the positions of the mutations in a three-dimensional model of hBD-3 compared with the wild-type protein. As shown in Fig 4C and 4D, in cancer tissue, mutant 2 is missing the third disulfide bond Cys63-Cys45 due to the Cys63→Leu mutation. We predict that this mutant will have a less stable structure than that of the wild type protein. S3 Table lists the effects of the other mutations. We have calculated the predictions of protein stability on the molecular structure of hBD-3 using both the PoP MuSiC [31] and CUPSAT [32] programs. Fig 3C and Fig 4C and 4D lists the observed frame-shift mutation types and the corresponding stabilizing/destabilizing effects on the hBD-3 structure. Mutations Cys63→Leu and Arg64→Pro affect amino acids that are buried in the structure and were determined to be energetically destabilizing, suggesting a reduction of protein stability. Mutations Arg66→Lys and Lys67→Pro affect solvent exposed residues and were determined to be energetically stabilizing (Table 3). Insertion 3 on exon 2 produced Lys67→Glu and the additional residue I68, which have no effect on the structure stability. Insertion 4–2 would produce a protein with an additional C-terminal leucine (Fig 3C).

**Table 3:** Predicted effect of the mutations affecting hBD-3 on protein structure stability.

Mutations	Predicted ∆∆G^a^ (Kcal/mol)	Solvent Accessibility^b^ (%)	Predicted ∆∆G^c^ (Kcal/mol)	Remarks
C63L	-2.41	0	-2.48	Buried residue. Leucine substitution destabilizing. Loss of SS bond.
R64P	-1.54	27.0	-2.13	Buried residue. Proline substitution destabilizing.
R65K	0.39	68.0	-0.13	Solvent exposed. Lysine substitution slightly overall stabilizing
K67E	0.26	100	0.14	Solvent exposed. Glutamate substitution stabilizing

^a^predicted protein thermal stability change (∆∆G in Kcal/mol) of mutation from CUPSAT program.

^b^relative solvent accessibility of the wild type residue computed from PoP MuSiC program.

^c^pridected protein stability change (∆∆G in Kcal/mol) of mutation from PoP MuSiC program.

Regarding hBD-4, insertion 1 may cause frame-shift mutations starting two residues after the signal peptide sequence, resulting in the inhibition of mature hBD-4 protein synthesis. Insertion 2 introduces a frame-shift mutation starting from residue 55 to 63, producing a truncated hBD-4 mutant protein

## Discussion

Colon cancer is a significant public health risk; it has been found to be the second most common malignancy in the Saudi population [33] and was ranked second in cancer death rates in the United States [34]. Many genetic and epigenetic mechanisms have been determined to contribute to colon cancer, including CpG hypermethylation, non-coding RNA alterations, somatic mutations and lysine acetylation of the histones [35]. Very few studies have focused on the epigenetic modifications responsible for the development of this disease. In the current study, we demonstrated for the first time a clear link between hBD expression/production and colon cancer. Our data demonstrated a significant reduction of hBDs in cancer tissues compared with normal tissues. These data are supportive of those previously reported with hBD-1in renal or prostate cancer [36–38] and also in oral squamous cell carcinoma (OSCC) [39,40]. Interestingly, similar data were reported with hBD-2, showing that hBD-2 mRNA and protein expression were significantly decreased in salivary gland cancer [41]. Other studies using different cancer cell lines have also demonstrated that hBD-2 may control cell growth via arrest of the G1/S transition and activation of pRB in malignant epithelial cells [42]. hBD-3 is known to suppress cancer cell migration [43]. In oral squamous cell carcinoma, human beta-defensin 1, 2 and 3 exhibit opposite effects on cell proliferation. Therefore, hBD-1 could be defined as a tumor suppressor gene, while hBD-2 and -3 might be proto-oncogenes [44].

The deregulation of gene expression is expected to affect 1–3% of the transcriptome [45], including innate immunity genes whose expression is predominantly down-regulated in cancer tissues. DNA methylation of CpG-rich promoter regions is gaining recognition as a key mechanism in the inactivation of the same genes involved as innate immunity and tumor suppressor genes in cancer cells [46]. Another mechanism for gene inactivation is somatic mutations. Theses mutations contribute to cancer formation. Our hypothesis was that down-regulation of hBD gene expression in colon cancer tissues may be due to the presence of epigenetic modifications, particularly mutations in the hBD exons. Instead, there was no detectable methylation in all the hBDs promoters of normal colon tissues and cancer colon cancer (data not shown). This may be due to the reduced number of CpG islands in hBD promoters. We have confirmed that in patients with a frame-shift mutation, the HBDs protein were completely absent. The growing interest in beta defensins is steadily enhancing our knowledge about various aspects of their gene location and expression patterns and the transcription factors involved in their regulation. The hBD genomic structure consists of two exons and one intron. The first exon encodes the signal peptide, and the second encodes a mature peptide preceded by a short anionic pro-peptide. In addition to having similar protein folding and structure, the structures of hBD-1, -2, and -3 have also revealed that each protein is stabilized by three disulfide bridges between conserved cysteines. In this study, many new mutations, generally insertions, were detected in different exons (1/2). These mutations contribute to significant changes in the protein structure of hBDs (i.e., hBD-1, hBD-3 and hBD-4). hBD-1 mutations and mutation 1 of hBD-3 are the most damaging because they lead to truncated pre-proteins with no predicted mature hBD-1 protein synthesis. hBD-3 mutation 2 protein is greatly destabilized because of the absence of a disulfide bridge caused by the substitution of Cys63→Leu. In addition, in the same mutant, Lys67→Glu substitution introduces a negatively charged, acidic residue in a positively charged, hydrophobic C-terminal section. As it was determined that the aggregation of positive charges at the C-terminal section is important for antimicrobial activity [47], the introduction of a negatively charged residue is predicted to affect the protein function. Both hBD-4 mutants are predicted to have no functional peptide because of changes in the protein sequence. No structure prediction could be performed for this mutant because its structure is not available. The other mutations found in the hBD promoter region are predicted to affect the expression/stability of the regulatory regions (5'UTR and 3'UTR) in colon cancer tissues, which explains why the expression of these hBDs were decreased in colon cancer tissues compared with normal tissues. Several other mutations were detected in the introns of all hBDs, which either primarily existed in all the tissues in the Saudi population (normal and cancer) or were only found in tissues from patients with colon cancer (data not shown). These intron mutations may have a possible role in colon cancer, and this finding requires further investigation. Because hBDs play an active role in the innate immune system, the presence of exon mutations in hBDs may lead to the dysregulation of innate immunity and subsequently hamper immune surveillance against colon cancer development.

## Materials and Methods

### General reagents

The RNA ladder was obtained from Ambion / Life technologies (Burlington, ON, Canada). DNA/ RNA kits were from Qiagen (Hilden, Germany). Primers were obtained from Life technologies/ Invitrogen (Burlington, ON, Canada). The high capacity cDNA reverse transcription kit was from Applied Biosystems (Warrington, USA). Sybr Green was obtained from Bio-Rad (Mississauga, ON, Canada). hBD antibodies were purchased from Santa Cruz Biotechnology, Inc (Santa Cruz, CA,U.S.A.). hBD-1 (N-20) is anti-Rabbit IgG for N terminal, hBD-2 (C-17) is anti-Goat IgG for N terminal. hBD-3 (FL-67) is anti-Rabbit IgG for N terminal. hBD-4 (FL-72) is anti-Rabbit IgG for N terminal. The Mega BACE kit for sequencing was obtained from GE Healthcare Life Science (Buckinghamshire, UK), and the EpiTect Bisulfite kit for DNA methylation was obtained from Qiagen (Toronto, Canada).

### Patient samples

Samples were collected from 40 patients diagnosed to have colon cancer (24 males and 16 females, between 45 and 75 years, with a mean of 60 ±16.56 years for males and 55 ±13.74 years for females and 40 age-matched normal controls with no cancer. The study was approved by the Institutional Review Board of the Ethics Committee at King Khalid University Hospital in Riyadh, KSA number 12/3352/IRB. Written informed consent was obtained from all participants.

Normal tissues in the distant margin to the tumor were collected at the time of endoscopy. The diagnosis of cancer was based on standard clinical, endoscopic, radiological, and histological criteria. Clinical and demographic characteristics were recorded, including age at diagnosis, gender, family history, smoking habits, disease behavior, disease location, and need for surgery (Table 1). The tissue samples were used for RNA extraction, immunohistochemistry, and genomic DNA extractions.

Tissue samples to be used for RNA analysis were immediately submerged in RNA later solution (Ambion, Courtabeuf, France).

### RNA isolation, reverse transcription and gene expression by real-time RT-PCR

Total tissue RNA was extracted using the DNA/RNA Mini kit (Qiagen, Hilden, Germany). The concentration, purity, and quality of the isolated RNA were all determined using the Agilent 2100 Bio-analyzer system and Agilent Small RNA analysis kit according to the instructions provided by the manufacturer (Agilent technologies, Waldbronn, Germany).

The RNA (1 μg of each sample) was reverse transcribed into cDNA using a high capacity cDNA reverse transcription kit (Applied Biosystems, Warrington, USA). The conditions for the preparation of the cDNA templates for PCR analysis were 10 min at 25°C, 2h at 37°C, and 5 min at 85°C. Quantitative PCR (qPCR) was performed as previously described [48–50]. The amounts of the mRNA transcripts were measured using the Applied Biosystems 7500 Fast real-time PCR detection system. Reactions were performed using a PCR Sybr Green Supermix from Applied Biosystems. Primers were added to the reaction mix at a final concentration of 250 nM. Five microliters of each cDNA sample were added to a 20-μl PCR mixture containing 12.5 μl of SYBR Green Supermix and 0.5 μl of specific primers (HBDs or GAPDH (S1 Table)) and 7 μl of RNase-/DNase-free water. Each reaction was performed in a 7500 fast real time PCR Thermal Cycler. The thermocycling conditions for the hBDs were established as 5 min at 95°C, followed by 36 cycles of 15 s at 95°C, 30 s at 63°C (except for HBD3 at 52°C), and 30 s at 72°C, with each reaction done in triplicate. The specificity of each primer pair was verified by the presence of a single melting temperature peak. GAPDH produced uniform expression levels varying by less than 0.5 CTs between sample conditions and was therefore used as a reference gene for this study. The amplified products were run on an agarose gel to confirm that there were no spurious products amplified during the cycles. The results were analyzed using the 2^-∆∆Ct^ (Livak) relative expression method.

### Preparation of biopsies for immunohistochemistry

Paraffin-embedded blocks of colon cancer tissue and normal colon tissue specimens were cut into 3-μm-thick sections. The sections were mounted on saline-coated slides and incubated for 15 to 20 minutes in a hot air oven at 60°C. Tissue sections were deparaffinized with EZ Prep (Ventana, Arizona, USA) at 75°C, heat pre-treated in Cell Conditioning 1 (CC1; Ventana, Arizona, USA) using a “standard cell conditioning” protocol for antigen retrieval at 100°C, and then incubated with one drop of inhibitor solution for four minutes at 37°C and subsequently washed. The slides were incubated for 32 minutes at 37°C with one of the following antibodies (diluted 1:100): anti-human-hBD-1, anti-human-hBD-2, anti-human-hBD-3 or anti-human-hBD-4 (Santa Cruz Biotechnology, CA, USA). Then, the secondary antibody ultraview universal HRP multimer was added. The immunolocalizedhBD-1, hBD-2, hBD-3 and hBD-4 proteins were visualized using a copper-enhanced DAB reaction. Negative blank controls were prepared during the staining, in which the first antibody was omitted and replaced with PBS. The slides were counter-stained with Hematoxylin II and Bluing Reagent (Ventana, Arizona, USA) for 30 min at 4°C, and then, liquid cover-slip (LCS) was added as a barrier between the aqueous reagents and the air to prevent evaporation, thereby providing sample stability. After that, the samples for mounting in DPX were dehydrated by sequential washes in graded alcohols: 70% ethanol, 96% ethanol and absolute ethanol and two changes in xylene. After that, we added a small drop of DPX to the specimen as amounting media. The immuno-stained sections were analyzed by an Olympus BX51 light microscope and DP72 Olympus digital camera (magnification 200X and 400X) (Olympus America Inc, Center Valley, PA, USA)**.**

Scores were given according to the level and the range of the color as follows: 0 points, no positive color; 1 point, <20% positive staining; 2 points, 21‑50% positive staining; 3 points, 51–75% positive staining; and 4 points, >75% positive staining.

### Bisulfite treatment

To check the methylation status of promoter region bisulfite treatment and recovery of samples were carried out with the EpiTect Bisulfite kit (QIAGEN) by following the manufacturer's instructions. Briefly, 2 μg DNA in 20 μL volume was used for each reaction and mixed with 85 μL bisulfite mix and 35 μL DNA protect buffer. Bisulfite conversion was performed on a thermocycler as follows: 99°C for 5 min, 60°C for 25 min, 99°C for 5 min, 60°C for 85 min, 99°C for 5 min, 60°C for 175 min and 20°C indefinitely. The bisulfite-treated DNA was recovered by EpiTect spin column and subsequently sequenced to confirm the efficiency of bisulfite conversion. The eluted genomic DNA was used for promoter region amplification and sequencing.

### Polymerase chain reaction and sequencing

Genomic DNA was extracted from all samples using a DNA kit from Qiagen (Hilden, Germany). The DNA concentration of each sample was measured using a NanoVue ultraviolet spectrophotometer. All the exons of the hBDs (1, 2, 3 and 4) were amplified using polymerase chain reaction (PCR) primers (S2 Table). The forward and reverse exon primer pairs used in the PCR reactions are described in S2 Table. The PCR mixture contained 50 ng DNA, 5 pmol/L each primer, 2.5nmol/mL each dNTP, and 1.25 U Taq DNA polymerase in 20 μL buffer with 0.04 μmol/L Mg2+. After an initial denaturation step at 94°C for 5 min, 35 PCR cycles were performed, which included 45 s for denaturation at 94°C, 30 s for annealing at 60°C, and 45 s for extension at 68°C. The PCR products were analyzed by 1% agarose gel electrophoresis. After observing clear and accurately sized bands, the amplification products were then purified and directly sequenced on a Sanger sequence detection system (GE Healthcare Bio-Sciences, Pittsburgh, PA, USA).

### Structural analysis of the mutations

The 3D structure of the human beta defensins (Protein Data Bank entry 1KJ6) [51] was used to estimate the impact of selected gene frame-shift mutations on the structure of the enzyme. The prediction of changes in thermal protein stability for each observed hBD-3 mutant was obtained from the CUPSAT and PoP MuSiC websites [31,32]. Both programs evaluated the change in free energy (i.e., the ΔΔ*G*) upon mutation of the protein folding-unfolding process. A positive or negative ΔΔ*G* value indicates that the mutation is thermodynamically stabilizing or destabilizing, respectively, while the magnitude of ΔΔ*G* indicates the extent of the alteration. The solvent accessibility of mutated residues was calculated using the PoP Music program.

### Quantification of staining and statistical analysis

The study was performed with 40 tumor samples and 40 controls, with the experimental values expressed as the means ± SD. The statistical significance of the difference between the control (normal tissues) and the test (tumor tissues) values was determined by means of a one-way ANOVA. For immunohistochemistry, positive staining was identified when the colon cancer tissues and normal tissues showed clear brown staining, and it was quantified by counting the positively marked cells Significant differences between individual groups were determined using an unpaired two way t-Test, with a P value < 0.05 considered statistically significant. Sequencing analysis was performed using DNASTAR (DNASTAR Inc, Madison, USA).

## Supporting Information

S1 Table Description of primer pairs used in real time PCR reactions. (DOC)

S2 Table Description of primer pairs used in PCR sequencing reactions for HBDs exons. (DOC)

S3 Table Predicted effect of the observed mutations on the hBD 1, 2, 3 and 4 gene product. (DOC)
