# Supplementary material for: Expression and New Exon Mutations of the Human Beta Defensins and Their Association on Colon Cancer Development
Source: PLoS One. 2015 Jun 3;10(6):e0126868. doi: 10.1371/journal.pone.0126868 (PMC4454434; doi:10.1371/journal.pone.0126868)
Supplement: S1 Table — (DOC) [file pone.0126868.s001.doc]

| **Gene** | **Primer sequence (5’ to3’ )** | **Amp size (bp)** | **Tm** |
| --- | --- | --- | --- |
| hBD-1 | Sense: 5’-GCCTCTCCCCAGTTCCTGAA--3  Antisense: 5’-GCAGAGAGTAAACAGCAGAAGGTA--3 | 82 | 63 °C |
| hBD-2 | Sense: 5’-TGTGGTCTCCCTGGAACAAAAT--3  Antisense: 5’-GTCGCACGTCTCTGATGAGG--3 | 105 | 63 °C |
| hBD-3 | Sense: 5’-CTTCTGTTTGCTTTGCTCTTCCT--3  Antisense: 5’-CTGTTCCTCCTTTGGAAGGCA--3 | 138 | 52 °C |
| hBD-4 | Sense: 5’-CACTCTACCAACACGCACCTAG--3  Antisense: 5’-CGCAACTGGAACCACACACT--3 | 133 | 63 °C |
| GAPDH | Sense: 5’-GGTATCGTCGAAGGACTCATGAC--3  Antisense: 5’-ATGCCAGTGAGCTTCCCGTTCAGC-3’ | 180 | 52°C to 63 °C |
