# Supplementary material for: Expression and New Exon Mutations of the Human Beta Defensins and Their Association on Colon Cancer Development
Source: PLoS One. 2015 Jun 3;10(6):e0126868. doi: 10.1371/journal.pone.0126868 (PMC4454434; doi:10.1371/journal.pone.0126868)
Supplement: S2 Table — (DOC) [file pone.0126868.s002.doc]

| **Gene/exon** | **Primer sequence ( to )** | **Size bp** | **Tm** |
| --- | --- | --- | --- |
|  |  |  |  |
| hBD-1/exon1 | FW: 5’-GCTCCAGGCTGAAAGCTAAA3’  RV: 5’- ATGCTTTCCTGCTGCTTGTT-3’ | 456 | 62 |
| hBD-1/exon2 | FW: 5’-ATCAATCATGGGCCAATTTC-3’  RV: 5’-GCGACAAGAGTGAAACACCA-3’ | 627 | 60 |
| hBD-2/exon1 | FW: 5’-GGGGTTTCCTGAGTCCAGAT-3’  RV: 5-GGAAGGGGAATGAGAGGAGAC-3’ | 369 | 60 |
| hBD-2/exon2 | FW: 5’-TGCCAGTTTCCATGTCAGAA-3’  RV: 5-ATGGGGAAGGTCAAGGAATC-3’ | 618 | 62 |
| hBD-3/exon1 | FW: 5’-GCCACATGCCCTGAGACTAT-3’  RV: 5’- GGTCCAAAGCACTCTGAAGG-3’ | 422 | 60 |
| hBD-3/exon2 | FW: 5’-GCTGCAGCCTTAGAAACATTG-3’  RV: 5- GCAAAGGGCATATTCCAAGA-3’ | 676 | 58 |
| hBD-4/exon1 | FW: 5’-TTCTGTAGCCCCAACACCTC-3’  RV: 5’-GGACCCAATGAAAGCAAAGA-3’ | 384 | 60 |
| hBD-4/exon2 | FW: 5’-GGTTGGGAATCAGAAGGTCA-3’  RV: 5-GAGTGGAAGCTCAGCAAACC-3’ | 475 | 60 |
