# Supplementary material for: Expression and New Exon Mutations of the Human Beta Defensins and Their Association on Colon Cancer Development
Source: PLoS One. 2015 Jun 3;10(6):e0126868. doi: 10.1371/journal.pone.0126868 (PMC4454434; doi:10.1371/journal.pone.0126868)
Supplement: S3 Table — (DOC) [file pone.0126868.s003.doc]

| Gene | Product |
| --- | --- |
| hBD3 | hBD3 peptide |
| Mutant 1 | No peptide |
| Mutant 2 | hBD3 peptide with C63L, R64P, R65K, K67E mutations + L68 |
| Mutant 3 | Peptide with K67E mutation |
| Mutant 4-1 | hBD3 peptide |
| Mutant 4-2 | hBD3 peptide + L68 |
